# Bcl-w Enhances Mesenchymal Changes and Invasiveness of Glioblastoma Cells by Inducing Nuclear Accumulation of β-Catenin

**DOI:** 10.1371/journal.pone.0068030

**Published:** 2013-06-27

**Authors:** Woo Sang Lee, Eun Young Woo, Junhye Kwon, Myung-Jin Park, Jae-Seon Lee, Young-Hoon Han, In Hwa Bae

**Affiliations:** 1 Division of Radiation Cancer Research, Korea Institute of Radiological & Medical Sciences, Seoul, Korea; 2 Department of Biological Sciences, Sookmyung Women’s University, Seoul, Korea; Sun Yat-sen University Medical School, China

## Abstract

Bcl-w a pro-survival member of the Bcl-2 protein family, is expressed in a variety of cancer types, including gastric and colorectal adenocarcinomas, as well as glioblastoma multiforme (GBM), the most common and lethal brain tumor type. Previously, we demonstrated that Bcl-w is upregulated in gastric cancer cells, particularly those displaying infiltrative morphology. These reports propose that Bcl-w is strongly associated with aggressive characteristic, such as invasive or mesenchymal phenotype of GBM. However, there *is no information* from studies of the role of Bcl-w in GBM. In the current study, we showed that Bcl-w is upregulated in human glioblastoma multiforme (WHO grade IV) tissues, compared with normal and glioma (WHO grade III) tissues. Bcl-w promotes the mesenchymal traits of glioblastoma cells by inducing vimentin expression via activation of transcription factors, β-catenin, Twist1 and Snail in glioblastoma U251 cells. Moreover, Bcl-w induces invasiveness by promoting MMP-2 and FAK activation via the PI3K-p-Akt-p-GSK3β-β-catenin pathway. We further confirmed that Bcl-w has the capacity to induce invasiveness in several human cancer cell lines. In particular, Bcl-w-stimulated β-catenin is translocated into the nucleus as a transcription factor and promotes the expression of target genes, such as mesenchymal markers or MMPs, thereby increasing mesenchymal traits and invasiveness. Our findings collectively indicate that Bcl-w functions as a positive regulator of invasiveness by inducing mesenchymal changes and that trigger their aggressiveness of glioblastoma cells.

## Introduction

Bcl-w (B cell lymphoma-w), is expressed in a variety of cancer types, including GBM and colorectal adenocarcinomas, as well as gastric cancers [1]. Above all, GBM is difficult to treat using the conventional therapeutic options of standard surgical resection, radiation and chemotherapy, owing to its high frequency of recurrence [2], as well as is related to the upregulation of Bcl-w [3], MMP-2 (matrix metalloproteinase-2) [4–7] and β-catenin [8]. This cancer type is highly proliferative and exhibits mesenchymal characteristics, leading to tumor progression through acquisition of invasive or metastatic potential. Growing evidence suggests that Bcl-w enhances not only survivability as a pro-survival member of the Bcl-2 (B cell lymphoma-2) protein family [9–11], but also the migratory and invasive potentials of cancer cells as an additional function. In an earlier investigation, we reported that Bcl-w enhances migratory and invasive potential in gastric cancer cells [12,13]. Additionally, nuclear accumulation of β-catenin is frequently observed in invasive cancer cells, which modulates downstream targets contributing to cancer stemness and malignancy by binding to TCF (T-cell factor) and LEF (lymphoid enhancer factor) in the nucleus [14]. However, there has been no currently available information about relationships between glioma cell characteristics and upregulated proteins, such as Bcl-w, MMP-2 and β-catenin. Based on the current findings, we conclude that Bcl-w is critical for malignancy by functioning as a positive regulator of mesenchymal traits and invasion, and contribute significantly to a more comprehensive understanding of the tissue-specific role of Bcl-w in GBM.

## Materials and Methods

### Cell culture, transfection, and treatments

The U251, U373, U87MG (glioma), MDA-MB-231 (breast cancer) and H1299 (lung cancer) were obtained from the Korean Cell Line Bank (KCLB). U251 cultured in Minimum Essential Medium Eagle (MEM) (Mediatech, Inc., Manassas, VA). U373, U87MG and MDA-MB-231 cultured in DMEM media (Mediatech, Inc., Manassas, VA). H1299 cultured in RPMI media (Mediatech, Inc., Manassas, VA) containing 10% FBS and penicillin-streptomycin antibiotics (PAA Laboratories GmbH, Pasching, Austria), respectively. The control and Bcl-w-overexpressing cells were transiently transfected with either empty pcDNA vector or that containing Bcl-w cDNA. Each experiment cells were transiently transfected with the indicated expression constructs or chemically synthesized small interfering RNAs (siRNAs; 20 nM) for 24 hours using Lipofectamine 2000 RNAi MAX (Invitrogen, Carlsbad, CA) (Invitrogen, Carlsbad, CA). The following small interfere RNAs purchased from; silencer negative control siRNA, si-Bcl-w-1 and si-Bcl-w-2 (Ambion, Cambridge, MA); si-vimentin, si-Twist1, si-Snail si-β-catenin, si-TCF-4, si-MMP-2 and si-FAK (Santa Cruz Biotechnology, Santa Cruz, CA).

### Antibodies and inhibitors

Antibodies were purchased from the following; two polyclonal anti-Bcl-w (goat) (R & D systems, Minneapolis, MN; Santa Cruz Biotechnology, Santa Cruz, CA); polyclonal anti-Twist (rabbit), monoclonal anti-Lamin A/C (mouse) and monoclonal anti-HA-probe (mouse) (Santa Cruz Biotechnology, Santa Cruz, CA); monoclonal anti-Snail (mouse) (Novus Biologicals, Littleton, CO); polyclonal anti-Slug (rabbit), polyclonal anti-p-Akt (rabbit), polyclonal anti-pGSK-3β (rabbit), polyclonal anti-p-β-catenin (rabbit) and monoclonal anti-TCF-4 (rabbit) (Cell Signaling technology, Beverly, MA); monoclonal anti-vimentin (mouse) (Thermo, Fisher Scientific, Fremont, CA); monoclonal anti-E-cadherin (mouse) and monoclonal anti-β-catenin (rabbit) (BD Transduction Laboratories, San Jose, CA); anti-β-actin (Sigma-Aldrich, St Louis, MO); polyclonal anti-GSK-3β (rabbit), monoclonal anti-FAK (mouse) and polyclonal anti-p-FAK (rabbit) (Invitrogen BioSource, Camarillo, CA); and monoclonal anti-MMP-2 (mouse) (Calbiochem, La Jolla, CA). The pharmacological inhibitors were used in this study; LY294002 (PI3K inhibitor) and Akt inhibitor (Calbiochem, La Jolla, CA).

### Western blot analysis

Proteins either in conditioned media or in cell lysates prepared using a previously described method [15] were separated by SDS-PAGE, and electrotransferred to Immobilon membranes (Millipore, Bedford, MA), which were subsequently blotted using the indicated antibodies and visualized by the ECL detection system (Amersham, Uppsala, Sweden).

### Immunofluorescence

5 Ⅹ10^4^ cells were seeded on coverslides (Paul Marienfeld GmbH & Co. KG, Lauda-Konigshofen, Germany). The cells were washed in filtered 1ⅩPBS and fixed with 4% paraformaldehyde (PFA) in 1ⅩPBS solution. The cells were permeabilized with 0.1% Triton X-100 in PBS. The cells were blocked with 5% normal goat serum (NGS) in 1ⅩPBS solution for 1 hour and then incubated with appropriate primary antibodies (1:200) for overnight at 4^o^C. The samples were reacted with Alex Fluor568 (red) or 488 (green)-conjugated secondary antibodies (1:200) for 1 hour at room temperature. Slides were mounted with Vectashield mounting medium solution containing DAPI (Vector Laboratories, Burlingame, CA) for 20 minutes at room temperature. Cells were visualized by confocal microscopy (Zeiss LSM710). Parameters of confocal microscopy are indicated here; Pinhole; Ch1: 60 µm, Ch2: 60 µm, Ch^3^: 60 µm, Lasers; 405 nm T1 4.5%, 488nm T2 7.0%, 543 nm T3 22.0%.

### Invasion assays

These assays were conducted as described previously [12]. In brief, to compare invasiveness, cells (2 × 10^5^) in 200 µl of medium were seeded onto the upper surfaces of Matrigel-coated polycarbonate filters which were placed in modified Boyden chambers (Corning, Corning, NY) that contain ECM components (BD Biosciences, Bedford, MA). The lower compartments of the chambers were filled with 1 ml of serum-free media supplemented with 0.1% BSA. After 20-24 hours of incubation at 37^o^C, the cells that had migrated to the lower surface of the filter were fixed and stained using a Diff-Quick kit (Fisher Scientific, Pittsburgh, PA), then counted under a microscope (Mitoti AE31 series, Trinocular inverted MIC) [16]. The results were analyzed for statistical significance using the Student’s *t*-test. Differences were considered to be significant at *p*< 0.05.

### Wound healing assays

Cell were harvested with buffered EDTA and plated into 12-well plates (6 × 10^4^ cells/well). The confluent monolayer were scratched after 24hours and then allowed to migrated for 24hours at 37°C. Cell in five fields in the scratched area (200 x 500 µm^2^ area) were counted under a light microscope (Mitoti AE31 series, Trinocular inverted MIC). Results were analyzed for statistical significance using Student’s *t* test. Differences were considered significant at *p*< 0.05.

### Gelatin zymography

This method was employed in order to analyze the activities of secreted MMPs. Conditioned media were prepared by incubating cells in serum-free media for 24 hours. Where indicated, the media were supplemented with specific inhibitors. Equal volumes of conditioned media were then subjected to 8% SDS-PAGE containing 0.1% gelatin. The gels were stained, and the MMPs activities were visualized as clear bands [16].

### Cell fractionation

After washing cells with PBS, cells gently suspend in buffer A (containing 10mM HEPES (pH 7.9), 10mM KCl, 3mM MgCl_2_, 0.5% NP-40 and protease inhibitors). Lyse the cells on ice for 30 minutes, centrifuge at 10000 rpm for 5 minutes. Repeat centrifugation of supernatant and take supernatant for cytoplasmic fraction. Wash the pellet with cold PBS twice and break nuclei in buffer B (containing 20mM HEPES (pH 7.9), 400mM NaCl, 3mM MgCl_2_, 0.2mM EDTA and 25% glycerol) on ice for 30 minutes. After centrifuge at 13000 rpm for 15 minutes, take the supernatant (nuclear fraction) apart from the insoluble pellets.

### Immunohistochemistry

To determine the relative levels of endogenous Bcl-w in normal (non-neoplastic) and human brain cancer tissues (WHO grades III/IV), premade AccuMax array brain cancer tissues with formalin fixed paraffin embedded slides (A221-IV; ISU Abxis CO. LTD., Seoul, Korea) were immunoreacted with anti-Bcl-w (R&D systems, Minneapolis, MN; 1:200). The levels of endogenous Bcl-w were stained with 3,3’-diaminobenzidine (DAB) substrate kit for peroxidase (Vector Laboratories, Burlingame, CA) after conjugation using avidin-biotin-peroxidase complex (Vector Laboratories, Burlingame, CA). Images were acquired by Olympus BX53F microscopy using cellSens Standard controller software (Olympus). The magnification is X100.

## Results

### Bcl-w is upregulated in glioblastomamultiforme tissues

**Figure 1:**
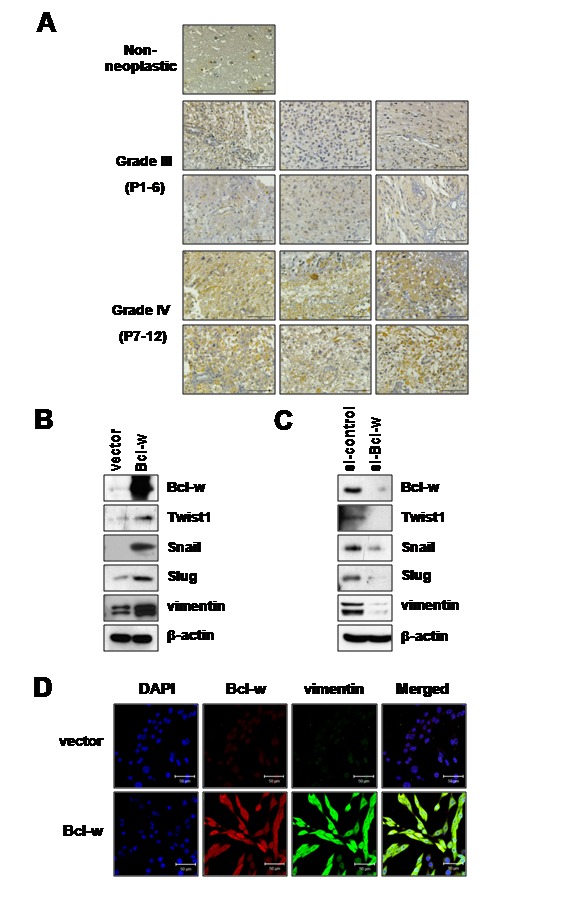
Bcl-w is upregulated in GBM and promotes the expression of mesenchymal-related marker proteins. **A**, premade brain cancer tissue microarrays (AccuMax, A221-iv) from patients were subjected to immunohistochemical staining with anti-Bcl-w antibody (R & D systems, Minneapolis, MN). Non-neoplastic and corresponding glial tumor grade iii/iv tissues from patients (P1-P12). Bar scale, 100 µm. **B**, U251 cells were transfected with either empty pcDNA vector or that containing Bcl-w cDNA. Bcl-w expression was detected using Western blotting with β-actin as the loading control. Expression levels of mesenchymal proteins were analyzed using Western blot analysis with anti-Twist1, anti-Snail, anti-Slug, anti-vimentin, anti-E-cadherin and anti-Bcl-w in control vector and Bcl-w-overexpressing cells. **C**, U251 cells were transfected with control or siRNA oligonucleotides targeting Bcl-w (20nM) for 24 hours. Transfected control or si-Bcl-w U251 cells were subjected to Western blot analysis with the indicated antibodies. **D**, confocal microscopy analysis of vector or Bcl-w-overexpressing cells showing Bcl-w (Red, Alexa 568) and vimentin (Green, Alexa 488) and DAPI (Blue). Scale bar, 50 µm.

Notably, Bcl-w expression was upregulated in GBM tissues (WHO grades IV), compared with normal and glioma grade III tissues in all patients examined (Figure 1A).

### Bcl-w positively regulates mesenchymal-related proteins

Epithelial-mesenchymal transition (EMT), characterized by loss of cell-cell adhesion, cytoskeleton rearrangement and manipulation of mesenchymal properties, is one of the crucial steps in cancer development, resulting in acquisition of the ability to metastasize. PI3K (phosphoinositide 3-kinase)/Akt and β-catenin/TCF-4 signaling pathways have been reported to play important roles in EMT and cancer progression [17–19]. In view of these data, we hypothesized that Bcl-w upregulates mesenchymal-related genes and plays a crucial role in acquisition of mesenchymal traits via stimulation of the PI3K-Akt-β-catenin-TCF-4 pathway.

Initially, we examined the expression levels of mesenchymal markers (vimentin, Twist1, Snail and Slug) in U251 glioblastoma cells via overexpression or depletion of Bcl-w with RNA interference (Figure 1B, C). Immunoblot analysis revealed that expression levels of vimentin, Twist1, Snail and Slug were markedly increased in Bcl-w-overexpressing cells, compared with control cells (Figure 1B). Bcl-w-targeted siRNA induced a significant decrease in levels of the mesenchymal protein markers vimentin, Twist1, Snail and Slug (Figure 1C). Immunofluorescence analysis additionally disclosed a marked increase in the expression of vimentin (Figure 1D) in Bcl-w overexpressing cells, compared with control cells. Other mesenchymal markers, N-cadherin and Claudin-1, were also increased in Bcl-w-overexpressing cells (data not shown). These results clearly demonstrate that Bcl-w promotes the expression of mesenchymal marker proteins, and thus contributes to the mesenchymal properties of GBM.

### Twist1 and Snail contribute to Bcl-w-induced invasiveness

**Figure 2:**
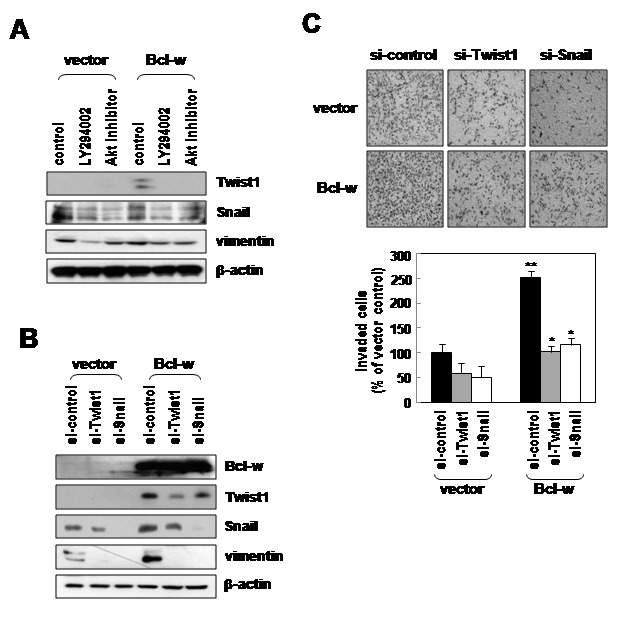
Twist1, Snail and vimentin regulate the invasiveness of glioblastoma cells. Down-regulation of Twist1 and Snail leads to inhibition of glioma invasion and vimentin expression. **A**, the indicated U251 cell transfectants were incubated in serum-free medium in the presence or absence of PI3K inhibitor (LY294002 (LY); 10 µmol/L) and Akt inhibitor (Akt-I; 10 µmol/L) for 1 hour. Expression levels of Twist1, snail and vimentin proteins were compared using Western blotting. **B**, control or Bcl-w-expressing U251 cells were transfected with 20nM of Twist1, Snail or vimentin siRNA for 24 hours and were subjected to Western blotting with mesenchymal-related proteins or anti-Bcl-w antibodies. **C**, cells in Figure 2B incubated in a Matrigel-coated transwell for 20 hours. *, *p*< 0.05, **, *p*< 0.005, n = 5.

Treatment of control vector or Bcl-w-expressing cells with a PI3K inhibitor (LY294002, 10 µmol/L) and Akt inhibitor (10 µmol/L) led to a significant decrease in Twist1, Snail and vimentinl levels (Figure 2A). Our data suggest that Bcl-w-induced mesenchymal marker proteins are regulated by PI3K-Akt-β-catenin signaling.

Treatment of control or Bcl-w overexpressing cells with Twist1 or Snail siRNA effectively decreased vimentin expression (Figure 2B) and Bcl-w-induced invasiveness (Figure 2C). Furthermore, vimentin-targeting siRNA reduced invasiveness in U251 cells (Figure S1).

### Bcl-w promotes migration and invasion in U251 glioblastoma cells

**Figure 3:**
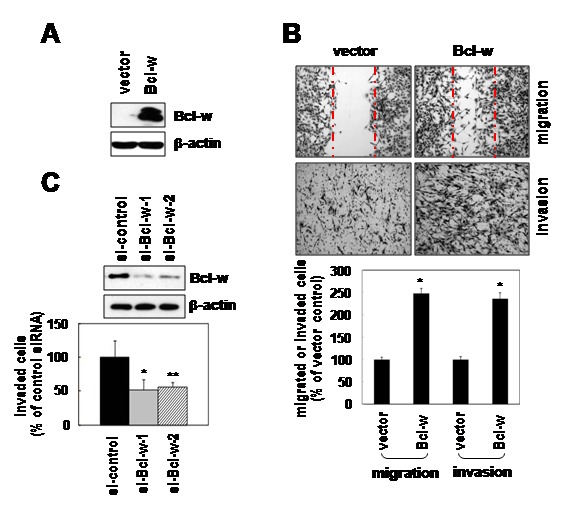
Bcl-w enhances migration and invasion in U251 glioblastoma cells. **A**, either empty pcDNA vector or that containing Bcl-w cDNA introduced into U251 cells. Bcl-w expression was detected using Western blotting. **B**, Bcl-w promotes migration and invasiveness of U251 glioblastoma cells. Top, the confluent cells in five fields from the scratched area (200 x 500 µm^2^) were counted under a light microscope. Transfectants were seeded onto Matrigel-coated polycarbonate filters to analyze their invasive potential. Cells were incubated for 20 hours in modified Boyden chambers, and the number of cells invading through filters stained and counted under a light microscope. Bottom, mean of triplicate experiments significantly different from controls. *, p< 0.05. **C**, two different siRNA sequences targeting Bcl-w (20nM of si-Bcl-w-1 and si-Bcl-w-2) were introduced into U251 cells for 24 hours, and the invasion assay conducted after 24 hours of incubation. Experiments were repeated five times, and the mean values and standard deviations determined. *, p< 0.05; **, *p*< 0.005.

We reported previously that Bcl-w enhances the migratory and invasive potential as well as survivability in gastric cancer cells [12,13]. Bcl-w expression has been positively associated with invading populations of cancer cells in GBM as well as gastric cancer. To establish the effects of Bcl-w on invasiveness and migratory ability in GBM, the protein was overexpressed in U251 cells (Figure 3A). Bcl-w-expressing cells displayed enhanced migration and invasion, as observed with the wound healing and invasion assay using Matrigel-coated polycarbonate filters, respectively (Figure 3B). To further ascertain the role of Bcl-w in invasiveness of U251 cells, the protein level was selectively reduced using two small RNA interference agents (siRNAs; si-Bcl-w-1 and si-Bcl-w-2). The Bcl-w-induced invasive ability of cells treated with these targeted siRNAs was effectively attenuated (Figure 3C).

### Bcl-w enhances the nuclear translocation of β-catenin

**Figure 4:**
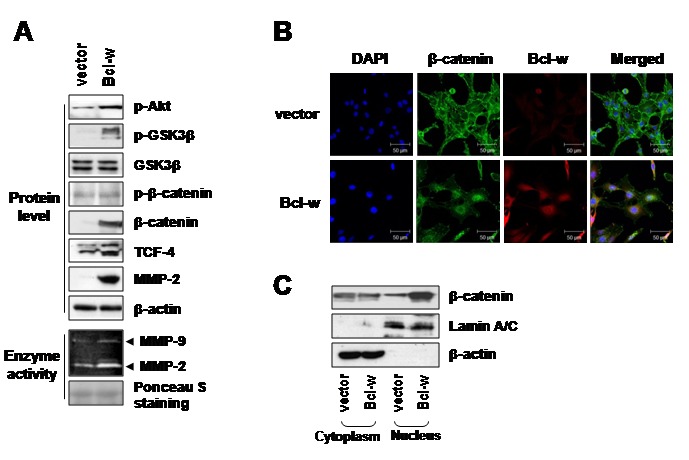
Bcl-w enhances transloation of β-catenin into the nucleus in U251 glioblastoma cells. **A**, levels of p-Akt, p-GSK3β, GSK3β, p-β-catenin, β-catenin and TCF-4 in cell lysates were compared by Western blotting using β-actin as a loading control. Conditioned media were prepared by incubating the vector and Bcl-w transfectants in serum-free medium for 24 hours. MMP-2 and MMP-9 activities were compared using zymography. Protein loading volumes were verified with Ponceau S staining. **B**, levels of β-catenin protein that translocated into the nucleus and Bcl-w protein in vector- or Bcl-w-transfected U251 cells were examined using confocal microscopy. Cells were stained with anti-β-catenin (green) or anti-Bcl-w (red) antibody, followed by nuclear staining with DAPI (blue). Scale bar, 50 µm. **C**, after separation of cells into cytoplasm and nuclear fractions for the indicated transfectants, each fraction was subjected to Western blotting with anti-β-catenin, anti-Lamin A/C (nucleus marker) and anti-β-actin (cytoplasm marker) antibodies.

We further investigated the molecular mechanisms underlying Bcl-w-induced cell migration and invasion. To this end, we showed that Bcl-w stimulates p-Akt, p-GSK-3β (glucose synthesis kinase-3β), β-catenin, TCF-4 and MMP-2 levels or activities using Western blotting and zymography analyses, respectively (Figure 4A). In particular, immunofluorescence and cell fractionation assays revealed phosphorylation of GSK-3β in Bcl-w-overexpressing cells in association with significantly enhanced nuclear accumulation of β-catenin, compared with control vector-transfected cells (Figure 4B, C). Based on these data, we suggest that Bcl-w regulates positively nuclear β-catenin translocation and its binding to TCF-4 in the nucleus, in turn, stimulating the expression of target genes, such as MMP-2 [20,21] to promote migration and invasiveness in U251 glioblastoma cells.

### PI3K and Akt act upstream of β-catenin in Bcl-w-induced invasiveness

PI3K-Akt signaling is a crucial regulator of tumor cell invasion, growth, proliferation, survival, metabolism and apoptosis under different experimental conditions [22]. Aberrant activation of the PI3K-Akt pathway and genetic alterations of its components contribute to tumorigenesis [23]. Notably, activation of PI3K-Akt signaling has been reported in 84% of GBM samples [24].

**Figure 5:**
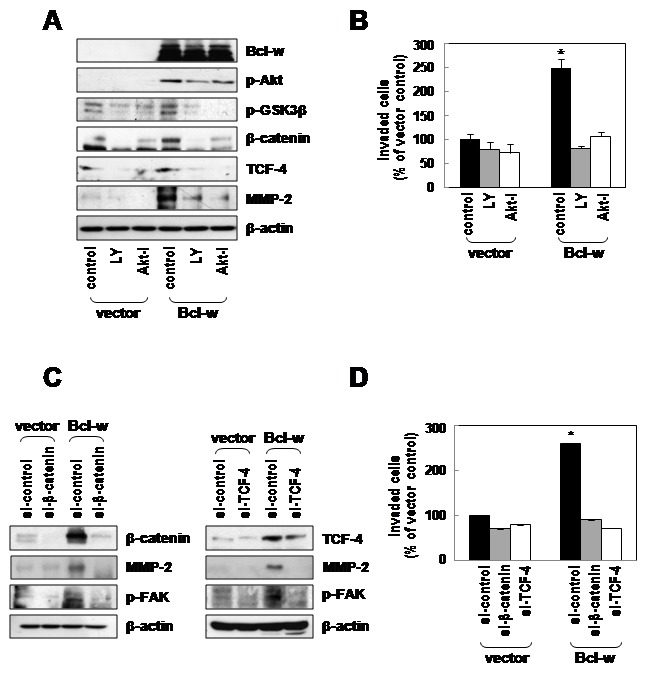
Bcl-w-induced β-catenin signaling components promote invasive potential via activation of the PI3K-Akt pathway. **A**, the indicated U251 cell transfectants were incubated in serum-free medium in the presence or absence of PI3K inhibitor (LY294002 (LY); 10 µmol/L) or Akt inhibitor (Akt-I; 10 µmol/L) for 1 hour. Expression levels and activities of p-Akt, p-GSK3β, β-catenin, TCF-4 and MMP-2 proteins were compared using Western blotting. **B**, cells treated with PI3K inhibitor or Akt inhibitor in the lower compartments of the invasion chambers for 24 hours, respectively. Invasive potential of treated cells was compared. *, p< 0.05 versus untreated control, n = 5. **C**, β-catenin and TCF-4 siRNAs (20nM) were introduced into vector or Bcl-w overexpressing cells, and cellular levels of β-catenin, TCF-4, MMP-2 and p-FAK compared after 24 hours of incubation using Western blotting with β-actin as a loading control. **D**, invasive potential of the indicated transfectants was compared. *, p< 0.05, n = 5.

To establish whether Bcl-w is linked to β-catenin via the PI3K-Akt pathway, control vector or Bcl-w-expressing cells were treated with an Akt inhibitor (10 µmol/L) and the PI3K inhibitor (LY294002, 10 µmol/L). The pharmacological inhibitors efficiently suppressed the expression of p-GSK3β, β-catenin, TCF-4 and MMP-2 proteins in cells displaying Bcl-w overexpression (Figure 5A), as well as Bcl-w-induced cell invasion (Figure 5B). Furthermore, siRNAs targeting β-catenin and TCF-4 induced a decrease in the MMP-2 and p-FAK (focal adhesion kinase) levels (Figure 5C), along with invasive potential (Figure 5D) of U251 cells. Our results indicate that PI3K and Akt act upstream of β-catenin in Bcl-w-induced glioblastoma cell invasion.

### MMP-2 and FAK signaling components are involved in Bcl-w-induced invasion

**Figure 6:**
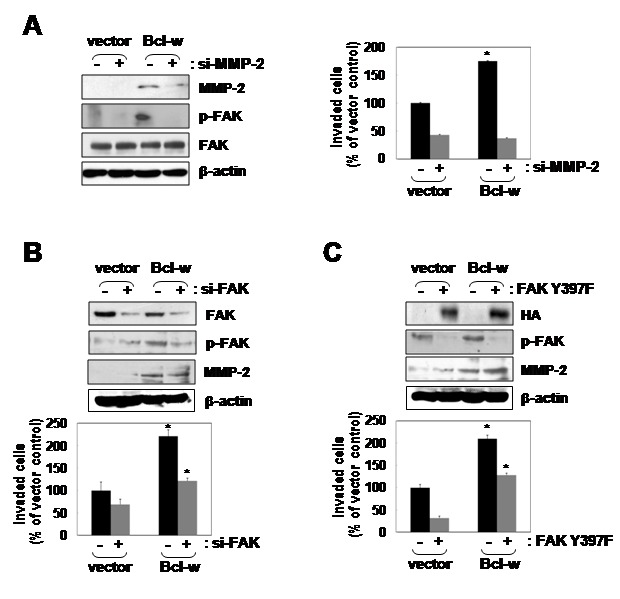
Activation of MMP-2 and FAK mediates Bcl-w-induced invasion upstream of FAK. **A**, right image, MMP-2 siRNA (20nM) was introduced into the indicated U251 transfectants, and after 24 hours of incubation, p-FAK (Y397) and MMP-2 protein levels were compared using Western blotting. Left image, invasion assays were performed using small interfering RNA MMP-2-treated and untreated cells. *, p< 0.01 versus untreated control, n = 5. **B**, top image, FAK siRNA was introduced into the indicated transfectants, and after 24 hours of incubation, cellular levels of FAK, p-FAK and MMP-2 compared using Western blotting. Bottom plots, invasion assays were conducted using the indicated cells. *, p< 0.05, n = 5. **C**, top images, vector- and Bcl-w-expressing cells were transiently transfected with expression vectors for HA-tagged dominant-negative FAK mutant (FAKY397F). After 24 hours of incubation, expression of the introduced mutants in cells was verified by Western blotting. Bottom plots, invasive potentials of the indicated cells were compared. *, *p*< 0.05, n = 5.

MMP-2-targeted siRNA markedly reduced phosphorylation of FAK and Bcl-w-induced invasiveness (Figure 6A). Moreover, suppression of FAK activity using RNA interference or dominant-negative mutants (FAKY397F) abolished Bcl-w-stimulated invasiveness (Figure 6B, C). Meanwhile, MMP2 levels are not changed by suppression of FAK. These data suggest that MMP-2 acts upstream of FAK in Bcl-w-induced glioblastoma invasion.

### Bcl-w promotes invasion in several cancer cell types

**Figure 7:**
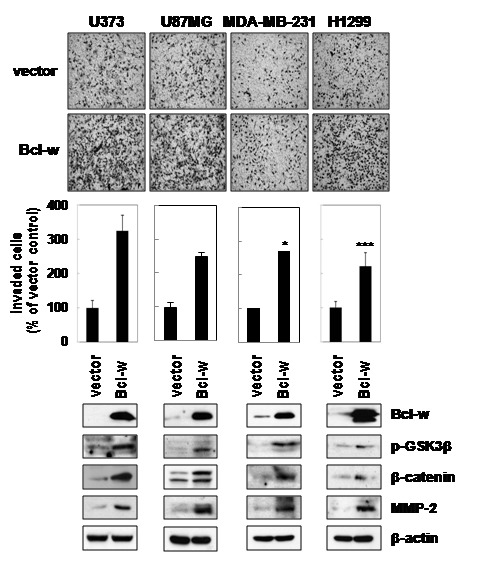
Bcl-w contributes to invasiveness in various cancer cell types including U373, U87MG, MDA-MB-231 and H1299. Cell lysates were prepared using vector- and Bcl-w-transfected cells, and Western blotting performed to compare p-GSK3β, β-catenin and MMP-2 protein levels. Transfectants were additionally subjected to Matrigel invasion assays. *, *p*< 0.01, ***, *p*< 0.0005 versus vector control, n = 5.

To determine whether Bcl-w plays an invasion-promoting role in general, we examined whether the protein enhances p-GSK3β, β-catenin and MMP-2 expression in several types of cancer cells, including U373, U87MG (glioma), MDA-MB-231 (breast cancer) and H1299 (lung cancer). Transient transfection with a control and Bcl-w-overexpressing vector was performed in various cancer cells. Overexpression of Bcl-w enhanced β-catenin and MMP-2 levels significantly through phosphorylation of GSK3β. Moreover, Bcl-w-overexpressing cells displayed elevated invasive ability (more than 1.5 to 3 fold), compared to those transfected with the control vector (Figure 7). To confirm these data using Bcl-w targeting siRNA, depletion of Bcl-w abolished invasive potentials in several cancer cells (Figure S2).

These results suggest that Bcl-w enhances the invasive potential and mesenchymal properties of glioblastoma cells via nuclear β-catenin accumulation, including activation of MMP-2 and FAK or expression of vimentin via activation of Twist1 and Snail, respectively (Figure S3).

## Discussion

We have previously reported that Bcl-w is upregulated in gastric cancer cells which has infiltrative morphology and enhances migratory and invasive potential in gastric cancer cells by inducing the production of several types of extracellular matrix (ECM)-degrading proteinases [12,13]. These reports propose that Bcl-w is strongly associated with aggressive characteristic of GBM which has invasive and mesenchymal phenotypes. However, there *is no information* from studies of the role of Bcl-w in GBM. To investigate the tissue-specific role of Bcl-w in GBM, data obtained from all the patients indicate that Bcl-w expression is upregulated in GBM tissues (WHO grades IV), compared with normal and glioma grade III tissues (Figure 1A). These results are supported by data that Bcl-w is upregulated in glioma tissues [3]. Recent reports have shown that glioblastoma cells have features of mesenchymal traits and invasiveness resulting in the tumor progression [25,26]. Loss of E-cadherin and gain of vimentin are considered major events, and Twist1, Snail and Slug are crucial transcription factors involved in the EMT process [27,28]. Twist1 enhances glioma invasion in concert with mesenchymal changes [29]. SNAI2/Slug is overexpressed in glioma cell lines and promotes invasion and growth in human gliomas [30]. SNAI1/Snail is involved in proliferation and migration of glioblastoma cells [31]. Our results show that Bcl-w promotes the expression of Twist1, Snail, Slug and vimentin proteins (Figure 1B). Immunofluorescence analysis additionally disclosed a marked increase in the expression of vimentin (Figure 1D) in Bcl-w overexpressing cells, compared with control cells. To confirm these data, transfection of U251 glioblastoma cells with Bcl-w-targeted siRNA significantly suppressed expression of the mesenchymal markers, Twist1, Snail, Slug and vimentin (Figure 1C), and reduced invasive ability via inhibition of MMP-2 production (Figure 6A). Similarly, Bcl-2 overexpression has been reported to induce partial EMT and promote squamous carcinoma cell invasion and metastasis [32]. Our data suggest that Bcl-w-induced mesenchymal marker proteins are regulated by PI3K-Akt-β-catenin signaling by treatment of PI3K inhibitor or Akt inhibitor (Figure 2A). Consistent with the theory that Bcl-w-induced signaling plays a key role in EMT events, depletion of Twist1/Snail led to reduced Bcl-w-induced vimentin expression and invasion, further confirming the association between mesenchymal factors and Bcl-w-induced invasive signaling (Figure 2B, C). Vimentin knockdown additionally inhibited invasion of U251 glioblastomas (Figure S1). These results provide strong evidence that Bcl-w reinforces mesenchymal changes as an important modulator of malignant progression in glioblastoma cells.

We further reported that upregulation of Bcl-w is associated with properties of GBM (such as mesenchymal traits and invasiveness), since Bcl-w overexpression increased migratory potential and invasiveness in glioblastoma U251 cells (Figure 3A–C). Previously, we demonstrated that Bcl-w-induced migratory and invasive properties are accompanied in gastric cancer cells [12,13]. However, our recent experiments have important meaning because of study about the role of Bcl-w related in the feature of this tissue. Bcl-w overexpression of GBM cells led to elevation of the levels of p-Akt p-GSK3β, β-catenin, TCF-4 and MMP-2 proteins (Figure 4A). Meanwhile, our previous study had showed that Bcl-w-induced Sp1 expression contributed to invasiveness by activating MMP-2 in gastric cancer cells. Therefore, we also investigated that Sp1 involved Bcl-w-induced invasion signaling in glioblastoma cells (data not shown; in submission). β-Catenin, such as another transcription factor which focused in this study, is a multifunction protein that participates in cell-cell interactions between E-cadherin and the cytoskeleton, and acts as an upstream regulator of TCF/LEF transcription factors. The constitutively activated Wnt/β-catenin signaling pathway is a key regulator in tumorigenesis and cancer malignancies [33–35], in particular, human gliomas [36,37]. Data from the immunofluorescence assay and cell fractionation using western blot analysis revealed that Bcl-w stabilizes the β-catenin level and facilitates its subcellular localization into the nucleus (Figure 4B, C) instead of cytoplasmic β-catenin degradation via the ubiquitin-proteosome system through formation of Axin-GSK3β-APC (adenomatous polyposis coli) complexes, by participating in the Wnt/β-catenin pathway. In the nucleus, β-catenin binds to LEF/TCF family of transcription factors, which may play a crucial role in the Wnt signaling pathway to drive the transcription of target genes, including cell proliferation, differentiation, and migration [38–40]. Our data suggest that Bcl-w-induced invasion of glioblastoma cells is related to the β-catenin/TCF-4 pathway that acts as a downstream regulator of PI3K and Akt (Figure 5A–D). Thus, β-catenin appears to participate in the invasion process. In addition, small interfering RNA against MMP-2 diminished activation of FAK and Bcl-w-induced invasion (Figure 6A). Finally, ablation of FAK activation with the use of siRNA or a dominant-negative mutant (Y397F) decreased invasion (Figure 6B, C). Meanwhile, MMP2 levels are not changed by depletion of FAK using siRNA or dominant negative mutant. These data suggest that MMP-2 acts upstream of FAK in Bcl-w-induced invasion of U251 cells. These data were supported previous data of gastric cancer cells, SNU484, that Bcl-w induced migratory and invasive potentials were enhanced the phosphorylation of FAK by activating of MMP-2 or uPA (urokinase plasminogen activator) [12,13].

Therefore, the invasion of glioblastoma stimulated by Bcl-w is mediated by a cellular signaling pathway that involves the translocation of nuclear β-catenin via activation of PI3K/Akt and sequential upregulation of MMP-2 and FAK signaling components. The promotion of invasive ability by Bcl-w appears to be a general phenomenon that occurs via similar signaling pathways in various cancer cell types (Figure 7, S2).

In summary, high expression of Bcl-w is clearly associated with mesenchymal changes and invading populations in the glioblastoma multiforme (Figure S3). Comprehensive investigation has revealed that Bcl-w functions as a positive regulator of invasion by enhancing mesenchymal traits of GBM, consequently contributing to malignancy. The findings of this study shed further light on the specific role of Bcl-w related in the features of glioblastomas.

## Supporting Information

Figure S1 Vimentin involves the invasive potentials of glioblatoma cells. U251 cells transfected with vimentin siRNA and vimentin expression in samples was confirmed using Western blotting. We additionally conducted the Matrigel-coated invasion assay after 20 hours. Invading cells were stained and observed using microscopy. ***, *p*< 0.0005, n = 5.
(TIF)

Figure S2 Bcl-w targeting siRNA attenuates invasiveness of U373, U87MG, MDA-MB-231 and H1299 cells. Bcl-w targeting siRNA introduced into U251 cells. Experiments were repeated five times, and the mean values and standard deviations determined. *, *p*< 0.05; **, *p*< 0.005, ***, *p*< 0.0005.
(TIF)

Figure S3 Schematic diagram of the Bcl-w-induced signaling pathway. Bcl-w promotes mesenchymal traits by inducing expression of vimentin via increasing the levels of Twist1 and Snail, transcription factors in the nucleus. In addition to, Bcl-w enhances the invasive ability of glioblastoma U251 cells by stimulating a pathway involving the sequential activation of PI3K, Akt, p-GSK3β, β-catenin and TCF-4, subsequently resulting in increased expression of MMP-2 and p-FAK. In conclusion, Bcl-w promotes mesenchymal traits and invasiveness by inducing the translocation of nuclear β-catenin and expression of target genes, such as vimentin or MMP-2 via increasing the levels of Twist1 and Snail in the nucleus.
(TIF)
