# Correction: Bcl-w Enhances Mesenchymal Changes and Invasiveness of Glioblastoma Cells by Inducing Nuclear Accumulation of β-Catenin

**DOI:** 10.1371/annotation/638b42e3-a351-4827-a612-17fe29b48e28

**Published:** 2013-08-27

**Authors:** Woo Sang Lee, Eun Young Woo, Junhye Kwon, Myung-Jin Park, Jae-Seon Lee, Young-Hoon Han, In Hwa Bae

The funding statement was omitted. The full funding information can be found below:

This work was supported by a grant from Nuclear Research & Development Program and Basic Science Research Program through the National Research Foundation of Korea (NRF) (NRF-2011-0013271) funded by the Korean Ministry of Education, Science and Technology. The funders had no role in study design, data collection and analysis, decision to publish, or preparation of the manuscript.

There was an error in the Figure 6 legend. The full, correct legend can be found below:

**Figure 6.** Activation of MMP-2 and FAK mediates Bcl-w-induced invasion upstream of FAK. **A**, left image, MMP-2 siRNA (20nM) was introduced into the indicated U251 transfectants, and after 24 hours of incubation, p-FAK (Y397) and MMP-2 protein levels were compared using Western blotting. Right image, invasion assays were performed using small interfering RNA MMP-2-treated and untreated cells. *, p< 0.01 versus untreated control, n = 5. **B**, top image, FAK siRNA was introduced into the indicated transfectants, and after 24 hours of incubation, cellular levels of FAK, p-FAK and MMP-2 compared using Western blotting. Bottom plots, invasion assays were conducted using the indicated cells. *, p< 0.05, n = 5. **C**, top images, vector- and Bcl-w-expressing cells were transiently transfected with expression vectors for HA-tagged dominant-negative FAK mutant (FAKY397F). After 24 hours of incubation, expression of the introduced mutants in cells was verified by Western blotting. Bottom plots, invasive potentials of the indicated cells were compared. *, p< 0.05, n = 5. 

