# Supplementary figures and images for: Bcl-w Enhances Mesenchymal Changes and Invasiveness of Glioblastoma Cells by Inducing Nuclear Accumulation of β-Catenin
Source: PLoS One. 2013 Jun 27;8(6):e68030. doi: 10.1371/journal.pone.0068030 (PMC3694904; doi:10.1371/journal.pone.0068030)

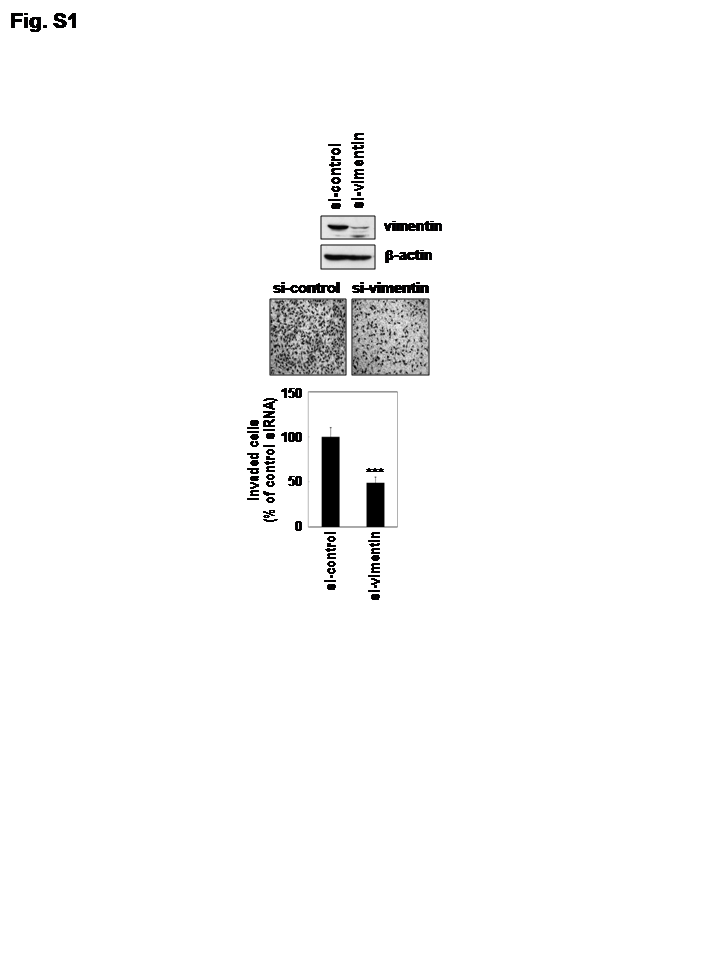

Supplement: Figure S1 — U251 cells transfected with vimentin siRNA and vimentin expression in samples was confirmed using Western blotting. We additionally conducted the Matrigel-coated invasion assay after 20 hours. Invading cells were stained and observed using microscopy. ***, p< 0.0005, n = 5. (TIF) [file pone.0068030.s001.tif]

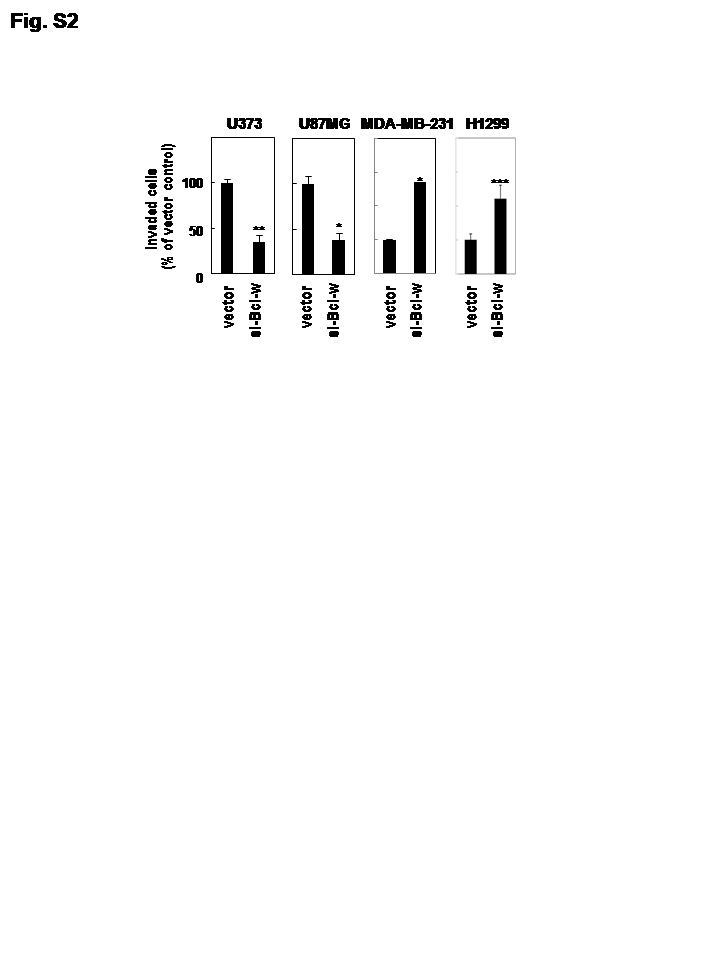

Supplement: Figure S2 — Bcl-w targeting siRNA introduced into U251 cells. Experiments were repeated five times, and the mean values and standard deviations determined. *, p< 0.05; **, p< 0.005, ***, p< 0.0005. (TIF) [file pone.0068030.s002.tif]

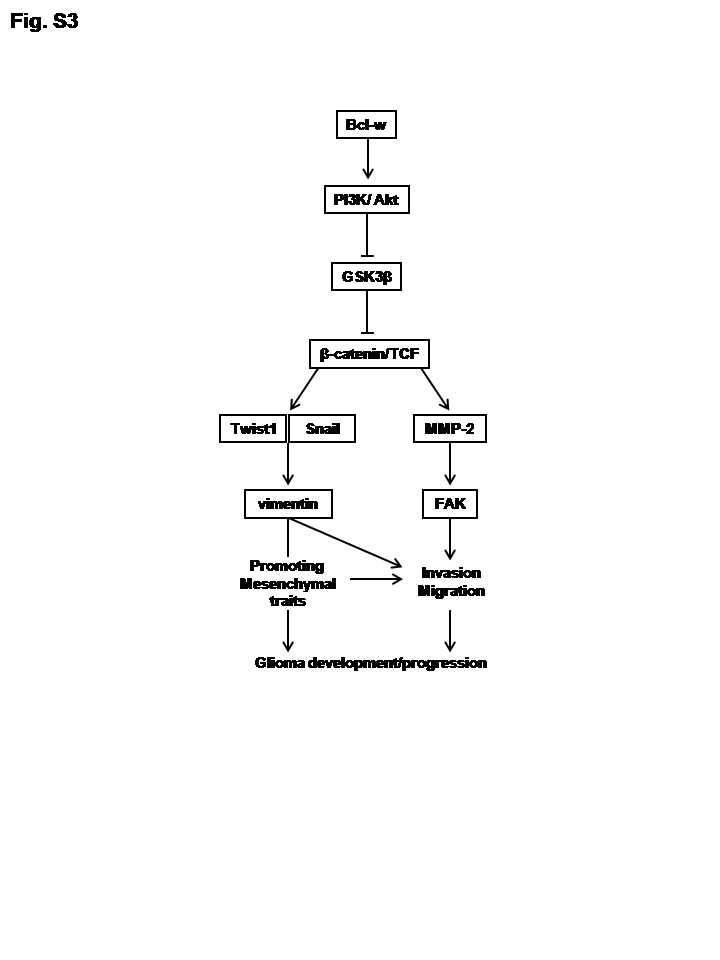

Supplement: Figure S3 — Bcl-w promotes mesenchymal traits by inducing expression of vimentin via increasing the levels of Twist1 and Snail, transcription factors in the nucleus. In addition to, Bcl-w enhances the invasive ability of glioblastoma U251 cells by stimulating a pathway involving the sequential activation of PI3K, Akt, p-GSK3β, β-catenin and TCF-4, subsequently resulting in increased expression of MMP-2 and p-FAK. In conclusion, Bcl-w promotes mesenchymal traits and invasiveness by inducing the translocation of nuclear β-catenin and expression of target genes, such as vimentin or MMP-2 via increasing the levels of Twist1 and Snail in the nucleus. (TIF) [file pone.0068030.s003.tif]
